# Axillary ultrasound and fine-needle aspiration in preoperative staging of axillary lymph nodes in patients with invasive breast cancer*

**DOI:** 10.1590/0100-3984.2014.0121

**Published:** 2015

**Authors:** Rafael Dahmer Rocha, André Ricardo Girardi, Renata Reis Pinto, Viviane Aguilera Rolim de Freitas

**Affiliations:** 1 MD, Radiologist, Trainee in Interventional Radiology at Hospital Israelita Albert Einstein, São Paulo, SP, Brazil.; 2 MD, Radiologist, Trainee in General Radiology at Med Imagem - Real e Benemérita Sociedade Portuguesa de Beneficência, São Paulo, SP, Brazil.; 3 MD, Breast Specialist, Hospital do Câncer III - Instituto Nacional de Câncer - Ministério da Saúde (INCA-MS), Rio de Janeiro, RJ, Brazil.; 4 MD, Radiologist, Hospital do Câncer III - Instituto Nacional de Câncer - Ministério da Saúde (INCA-MS), Rio de Janeiro, RJ, Brazil.

**Keywords:** Ultrasound, Fine-needle aspiration, Axillary lymph node, Breast cancer

## Abstract

**Objective:**

To propose an algorithm to determine the necessity for ultrasonography-guided fine-needle aspiration (US-FNA) in preoperative axillary lymph node staging of patients with invasive breast cancer.

**Materials and Methods:**

Prospective study developed at National Cancer Institute. The study sample included 100 female patients with breast cancer referred for axillary staging by US-FNA.

**Results:**

The overall US-FNA sensitivity was set at 79.4%. The positive predictive value was calculated to be 100%, and the negative predictive value, 69.5%. The US-FNA sensitivity for lymph nodes with normal sonographic features was 0%, while for indeterminate lymph nodes it was 80% and, for suspicious lymph nodes, 90.5%. In the assessment of invasive breast tumors stages T1, T2 and T3, the sensitivity was respectively 69.6%, 83.7% and 100%. US-FNA could avoid sentinel node biopsy in 54% of cases.

**Conclusion:**

Axillary ultrasonography should be included in the preoperative staging of all patients with invasive breast cancer. The addition of US-FNA in cases of lymph nodes suspicious for malignancy may prevent more than 50% of sentinel lymphadenectomies, significantly shortening the time interval to definitive therapy.

## Abstract (pt)

**Objetivo:**

Propor um algoritmo de quando a punção aspirativa por agulha fina guiada por ultrassonografia (PAAF-US) deve ser utilizada no estadiamento linfonodal axilar pré-operatório em pacientes com câncer de mama invasivo.

**Materiais e Métodos:**

Estudo prospectivo conduzido no Instituto Nacional de Câncer. Participaram da amostra 100 pacientes do sexo feminino portadoras de câncer de mama que foram direcionadas para estadiamento axilar por meio de PAAF-US.

**Resultados:**

A sensibilidade geral da PAAF-US foi estabelecida em 79,4%. O valor preditivo positivo foi calculado em 100% e o valor preditivo negativo, em 69,5%. A sensibilidade da PAAF-US para linfonodos com características ultrassonográficas normais foi 0%, ao passo que para os indeterminados foi 80% e para os suspeitos foi 90,5%. Na avaliação de tumores de mama invasivos estádios T1, T2 e T3, as sensibilidades foram 69,6%, 83,7% e 100%, respectivamente. A realização da PAAF-US conseguiu evitar a biópsia do linfonodo sentinela em 54% dos casos.

**Conclusão:**

A ultrassonografia axilar deve estar inclusa no estadiamento pré-operatório de todas as pacientes com câncer de mama invasivo. A adição da PAAF-US nos linfonodos com características morfológicas suspeitas de malignidade pode evitar mais de 50% das linfadenectomias sentinelas, proporcionando abreviação importante do intervalo de tempo até a terapêutica definitiva.

## INTRODUCTION

Malignant breast neoplasia is the main cause of deaths caused by cancer in women worldwide. The introduction of new techniques has allowed the diagnosis of early-stage lesions and more conservative treatments. Currently, the histopathological diagnosis of breast cancer is carried out by means of minimally invasive methods, whenever possible by means of imaging-guided percutaneous biopsy^(1)^. On its turn, the evaluation of axillary lymph node staging constitutes one of the most relevant prognostic indicators for breast cancer patients^(2-8)^, as the axilla is the receptor of approximately 95% of the breast lymphatic drainage^(9,10)^. Therefore, axillary lymph node dissection has been considered, for many years, as the gold standard method in the diagnosis and treatment of lymph node metastases.

Over the past decades, other methods have been utilized in the prediction of axillary lymph node positiveness, such as sentinel lymph node biopsy (SLNB) and ultrasonographyguided fine needle aspiration biopsy (US-FNA). First practiced by Krag et al.^(11)^ in 1993, SNLB demonstrated to be equivalent to axillary lymphadenectomy, with expressive reduction of morbidity rates^(12)^. However, it is also invasive and time consuming procedure, with possible complications. Because of this, US-FNA emerged as a faster method with very low complication rates. In cases of patients with positive results at US-FNA, the investigation of the sentinel lymph node can be avoided and the patient can be directly referred for axillary lymph node dissection, or even to neoadjuvant chemotherapy (CT)^(4,7,13,14)^.

In spite of potential advantages of US-FNA over SLNB, some institutions have not routinely adopted US-FNA for initial axillary staging in cases of breast cancer^(4)^. Some authors recommend it only for primary tumors > 1.0 cm. The alleged justification is that the signs of axillary lymph node involvement in smaller invasive primary breast tumors would be less defined^(15,16)^.

The present study was aimed at proposing an algorithm to define when US-FNA should be utilized in the preoperative axillary lymph node staging in patients with invasive breast cancer.

## MATERIALS AND METHODS

### Study design

With research ethics board approval the study was conducted at the Breast Radiology Department of Hospital do Câncer III - Instituto Nacional de Cancer. Female patients presenting with histopathologically confirmed invasive breast cancer - either by means of percutaneous biopsy or surgical biopsy - which had been referred for axillary staging by means of US-FNA, participated in the study.

Only those patients who were candidates for SNLB and who presented with tumor staging (TNM classification)^(17)^ up to T3, and clinically negative axillae were included in the study. The patients were referred by mastologists and radiologists of the mentioned Institution, in cases where there were doubts on lymph node compromising at clinical examination (enlarged but not adherent lymph nodes) or presence of suspicious morphological lymph node change at any imaging study (mammography, ultrasound or magnetic resonance imaging).

Exclusion criteria were the following: presence of multifocal or multicentric tumors; patients with previous history of surgery, chemotherapy or radiotherapy treatment for the respective cancers; patients who were not submitted to surgical axillary evaluation afterwards (either SNLB or axillary lymph node dissection); and patients whose cytopathological analysis suggested the presence of lymph node metastasis of histopathological subtype different from breast cancer.

The first 100 patients who met the above mentioned criteria were prospectively selected in the period from January/2011 to August/2013. All patients were assessed by ipsilateral axilla US of the tumor, with morphological characterization of the lymph nodes and performance of US-FNA at the same moment.

The cytopathological results were compared with the histopathological results obtained from SNLB or from axillary lymph node dissection, which were considered the gold standard. Initially, the patients with negative or insufficient cytopathological results were first submitted to SLNB, by means of peripapillary radiopharmaceutical injection. Those patients with positive cytopathology were directly referred to axillary lymph node dissection or neoadjuvant CT. The cytopathological analyses were performed by three pathologists with at least five-year experience.

### Selection and characterization of the lymph node at US

**Figure 1:**
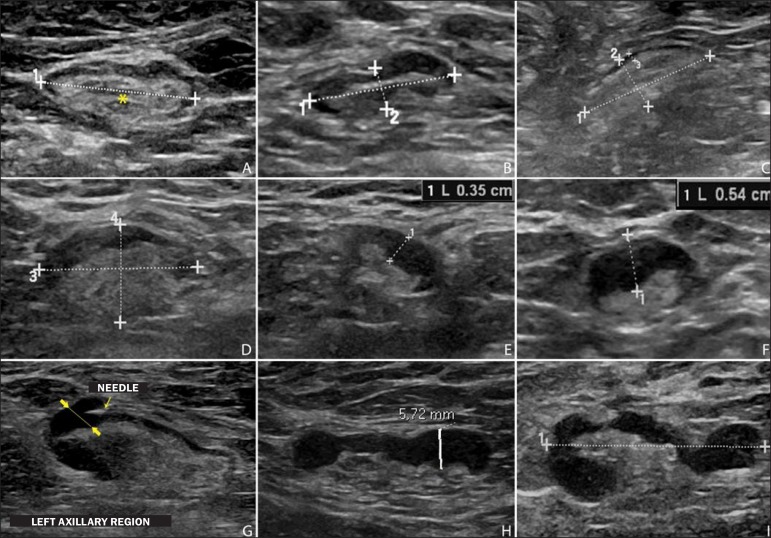
Sonographic images demonstrating some lymph nodes classified as morphologically normal (**A-C**) and indeterminate (**D-I**). Normal lymph nodes characteristically present with central fatty hilum (asterisk) and diffuse cortical thickening ≤ 3 mm. The indeterminate lymph nodes presented with central hilum, however with some area with cortical thickening > 3 mm (between arrows). The **A-C** lymph nodes demonstrated negative histopathological results, while the **D-I** lymph nodes were positive.

**Figure 2:**
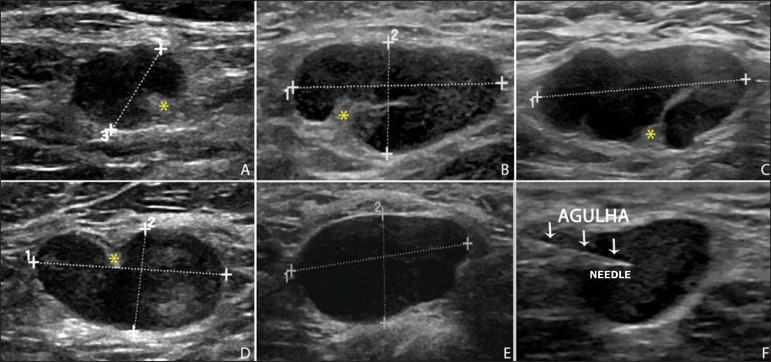
Sonographic images demonstrating some lymph nodes morphologically classified as suspicious. The **A-D** lymph nodes present with marked cortical thickening, determining replacement and marginalization of the fatty hilum (asterisks). In more advanced cases some lymph nodes may present with total absence of the hilum (**E,F**)

The US-FNAs were performed by two authors (R.D.R. and R.R.P) with at least two-year experience in the procedure. By utilizing a high-frequency linear transducer (11 MHz) and a GE Logic E9^®^ apparatus, one sought to identify the lymph nodes with morphological changes, so the aspirate was obtained from only one of them, according to criteria of decreasing suspicion: a) lymph node with absent hilum; b) lymph node with cortical thickening > 3 mm and eccentric hilum (peripheral); c) lymph node with any area of cortical thickening > 3 mm and central hilum. The thickness of the lymph node cortex was always measured at its thickest portion. The "a" and "b" lymph nodes were classified as suspicious, while the "c" lymph nodes were classified as indeterminate. In the absence of any suspicious change, US-FNA was performed in morphologically normal lymph nodes identified at the most inferior axillary level. Figures 1 and 2 show examples of some lymph nodes from each category of the mentioned sonographic morphological classification.

### US-FNA procedure

Initially the patients were explained about the reason for the procedure, the procedural technique, risks and benefits, existence of alternative techniques, and then they are asked to sign a term of free and informed consent. Next, asepsis was performed in the axillary region, and anesthesia was applied to the skin (about 3 mL lidocaine at 2%). The puncture was performed with a 21-gauge needle on a 10 mL syringe. In order to obtain the cytological material, the needle was moved in various directions (fan shaped movements) maintaining vacuum that was undone before removal of the needle. In the lymph nodes with focal cortical thickening, preferably the aspiration was performed in the altered region (Figure 1G). A sonographic image was acquired showing the tip of the needle within the target (Figure 2F). Enough aspirates were obtained to prepare two slides, which were fixed with 95.6% ethanol, and later sent for cytological analysis.

### Statistical analysis

For the US-FNA procedures, as well as for axillary US alone, the sensitivity and specificity rates were calculated, as well as positive predictive value, negative predictive value and accuracy. The sensitivity of US-FNA was also calculated according to the lymph node morphology at axillary US and size of the primary tumor (T stage). The factors associated with axillary lymph node compromising and with increase in US-FNA sensitivity were also estimated. Finally, a percentage of avoided SNLB was established for the patients in the sample. The software utilized for such analyses was the Epi Info 7^®^.

In the estimation of risk variables associated with axillary lymph node positiveness and US-FNA sensitivity, the following factors were taken into consideration for the purposes of univariate logistic regression analysis: age; absent or eccentric hilum; cortical thickening > 3 mm; stage ≥ T2; longitudinal diameter ≥ 2.0 cm; transverse diameter ≥ 1.0 cm; longitudinal/transverse diameter ratio < 1,5; estrogen receptor positiveness; progesterone receptor positiveness; and Her-2 receptor positiveness. Only the variables with values of *p* < 0.1 were included in the multivariate analysis. The variables risk estimation was expressed in odds ratio (OR) with 95% confidence interval (95% CI). Statistical significance was set as *p* < 0.05.

## RESULTS

The demographic data and tumor characteristics of the sample are shown on Table 1. The mean age of the patients was 53.7 years (interval from 27 to 86 years). Ductal carcinoma was the most prevalent histological tumor type identified in 80% of the cases. The most frequent tumor measurement established by means of mammographic images review or US measurement concomitant with US-FNA was between 2.0 and 5.0 cm (stage T2). Only 3% of the patients presented with tumors > 5.0 cm (T3). Once the patients submitted to neoadjuvant CT were excluded, the mean time interval between US-FNA and surgery was 65 days (interval of 18 to 185 days).

**Table 1:** Demographic data and tumor characteristics of 100 patients submitted to ultrasonography-guided fine needle aspiration biopsy.

Characteristics	Percentage of patients
Mean age (age range)	53.7 years (27-86 years)
Cancer laterality	
Left breast	58%
Right breast	42%
Histological tumor type	
Ductal	80%
Lobular	3%
Mixed	9%
Other	8%
Radiological T stage	
T1	37%
T2	60%
T3	3%
Pathological T stage	
T0 (not identified)	6%
T1	41%
T2	49%
T3	4%
Pathological N stage	
0	38%
1	30%
2	17%
3	15%
Neoadjuvant chemotherapy	14%
Estrogen-receptor positiveness	80%
Progesterone-receptor positiveness	71%
Her-2 receptor positiveness	15%

### US-FNA performance and its correlation with morphological lymph nodes characteristics and primary tumor size

**Figure 3:**
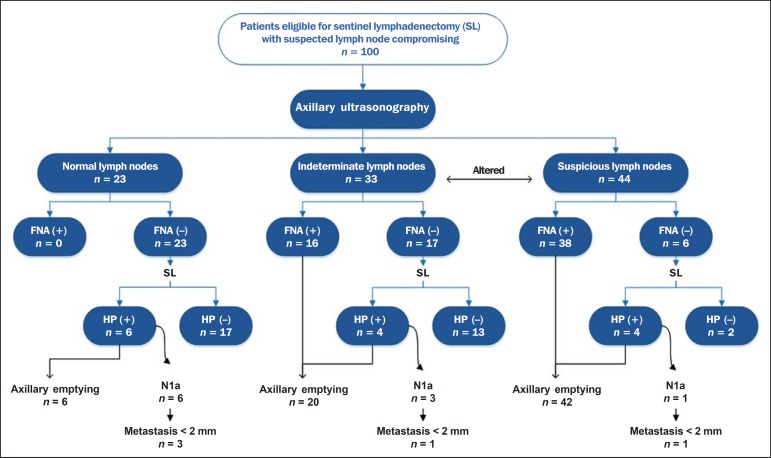
Flowchart of patients submitted to axillary ultrasonography and lymph node fine needle aspiration (FNA) biopsy according to morphological characterization and correlation with histopathological (HP) results.

The histopathological positiveness of lymph nodes in the sample was 68%. In 18 cases (29% of compromised axillae) only one positive lymph node was found at axillary emptying. The flowchart of patients submitted to US-FNA is represented on Figure 3.

The US-FNA results considered to be positive in 54 patients (54%), negative in 38 patients (38%) and insufficient in 8 patients (8%). For statistical purposes, the insufficient samples were grouped with the negative ones, as they did not avoid the sentinel lymphadenectomy approach.

Total sensitivity of US-FNA in the sample was established in 79.4% (54/68). The sensitivity of US-FNA for lymph nodes considered as normal was 0% (0/6), while for those considered as indeterminate it was 80% (16/20), and for the suspicious ones it was 90.5% (38/42). On Table 2, one can observe the US-FNA sensitivity according to morphological lymph node characteristics and size of primary tumor (stage T of the TNM classification). As indeterminate and suspicious lymph nodes were considered as a single group ("altered lymph nodes"), the US-FNA sensitivity was calculated as being 87.1% (54/62).

**Table 2:** US-FNA sensitivity according to morphological lymph node characteristics and primary tumor size.

	US-FNA sensitivity (number of US-FNA / positive histology / positive US-FNA)						
Lymph nodes	T1a	T1b	T1c	T1	T2	T3	Total
Normal	0%	0%	0%	0%	0%	—	0%
	(3 / 0 / 0)	(1 / 0 / 0)	(9 / 5 / 0)	(13 / 5 / 0)	(10 / 1 / 0)	(0 / 0 / 0)	(23 / 6 / 0)
Indeterminate	100%	0%	66.6%	75%	78.6%	100%	80%
	(3 / 1 / 1)	(2 / 0 / 0)	(3 / 3 / 2)	(8 / 4 / 3)	(22 / 14 / 11)	(3 / 2 / 2)	(33 /20 / 16)
Suspicious	50%	100%	100%	92.8%	89.2%	—	90.5%
	(3 / 2 / 1)	(2 / 2 / 2)	(11/ 10 / 10)	(16 / 14 / 13)	(28 / 28 / 25)	(0 / 0 / 0)	(44 / 42 / 38)
Total	66.6%	100%	66.6%	69.6%	83.7%	100%	79.4%
	(9 / 3 / 2)	(5 / 2 / 2)	(23 / 18 / 12)	(37 / 23 / 16)	(60 / 43 / 36)	(3 / 2 / 2)	(100 / 68 / 54)

No false-positive result from US-FNA was identified, characterizing 100% specificity and positive predictive value. On the other hand, US-FNA produced 14 false-negative results, determining a negative predictive value of 69.5%. Ten out of those patients, (71.4%) presented with only 1 to 3 compromised lymph nodes (lymph node staging N1a). It can also be observed that in 5 cases (35.7%), only lymph node micrometastases (< 2 mm) were found. The US-FNA accuracy was estimated to be 86%.

Neoadjuvant CT was instituted in 14 (25.9%) of the 54 patients with positive cytopathological results. No neoadjuvant CT was instituted for patients with negative US-FNA. At least 10 patients (71.4%) had histopathological reports indicating signs of response to CT (fibrosis, sclerosis, hyalinization, lympho-plasmacytic infiltrate); among those, 6 (42.8%) were considered as complete response, i.e., without evidence of tumor lesion. Also, one observed that neoadjuvant CT could effectively improve tumor stage (T) in 64.2% of cases (9/14), with two complete responses. All six cases with complete lymph node response to neoadjuvant CT presented optimal response in the primary lesion.

The US-FNA sensitivity for tumors stages T1, T2 and T3 was 69.6%, 83.7% and 100%, respectively.

### Factors associated with malignancy and increased US-FNA sensitivity

At logistical regression and multivariate analysis, the following risk factors were associated with lymph node compromising: presence of cortical thickening > 3 mm (OR = 3.9; 95% CI: 1.13-13.3; *p* = 0.03); absent or eccentric hilum (OR = 9.23; 95% CI: 1.76-48.3; *p* = 0.008). Transverse diameter ≥ 1.0 cm was the sole factor associated with increased US-FNA sensitivity in the sample (OR = 5.68; 95% CI: 1.08-29.8; *p* = 0.04).

### Axillary US alone

The sensitivity of axillary US alone was 91.2% (62/68), with positive predictive value of 80.5% (62/77). Specificity was 53.1% (17/32) and the negative predictive value was 73.9% (17/23). The accuracy of axillary US alone was estimated to be 79% (79/100).

### Avoided sentinel lymph nodes investigation

The utilization of US-FNA avoided SLNB in 54% of patients in the sample. When analyzing only US-FNA from patients presenting with altered lymph nodes (either suspicious or indeterminate), one could avoid investigation of sentinel lymph nodes in 70.1% of cases (54/77).

## DISCUSSION

The Brazilian radiological literature has recently demonstrated interest on the sonographic evaluation of axillary lymph nodes^(18)^, mainly whether US-FNA is an effective method to predict lymph node compromising in breast cancer patients^(19)^. Although the utilization of axillary US in association with fine needle aspiration is capable of avoiding a great number SLNB, there is no consensus about when it should be indicated. Among the main arguments involving the subject, one should highlight the difficulty in defining whether axillary US should be performed in all patients presenting with a primary invasive breast tumor or only in those cases of tumors above a certain size. Many authors support the indication of axillary US for all breast cancer patients, independently of tumor size^(4,20-23)^. On the other hand, Mainiero et al.^(13)^ and de Kanter et al.^(24)^ recommend axillary US only in cases of tumors > 1.0 cm.

The present study has demonstrated that the US-FNA sensitivity increased in a directly proportional relation with the primary tumor size, as previously demonstrated by Koelliker et al.^(4)^, Mainiero et al.^(13)^ and Somasundar et al.^(16)^. The lymph node positiveness rate observed in patients in stage T1 (< 2,0 cm) was 62.1%, while for the sub-group T1a and T1b (< 1,0 cm) it was 35.7%. It was also observed that 71.5% of the patients with tumors < 1.0 cm presented with morphologically altered lymph nodes, with US-FNA sensitivity being calculated to be 80% in this subgroup. Even with only 10-30% of the patients with tumors < 2.0 cm presenting with axillary involvement^(25-27)^, the above described results suggest that the most important predictive factors for malignancy and US-FNA positiveness are morphological lymph node alterations, independently of primary tumor size. A study with a larger sample in this population (stage T1) is required, as the need for axillary emptying has been under discussion for patients with invasive carcinoma even in cases where sentinel lymphadenectomy is positive in this specific subgroup^(28)^.

Another question involves whether US-FNA should be performed in all lymph nodes, regardless the presence or not of morphological changes. Some authors recommend the utilization of US-FNA as a routine in the initial approach^(20,29)^. In the present study, no positive result was obtained in USFNA of morphologically normal lymph nodes. Also, it was observed that in 6 out of the 14 false-negative results no morphologically altered lymph node was observed at US. Lymph node micrometastases were found in 35.7% of the false-negative cases, a rate that is similar to the ones reported in other studies^(4,7,10,20,30)^.

As the possible malignancy predictors were evaluated, one observed that sonographic findings demonstrating cortical thickening > 3 mm (especially ≥ 6 mm) and change in the fatty hilum were strongly associated with malignancy, in agreement with data reported by other studies^(4,21,31)^. According to Deurloo et al.^(21)^ and Mainiero^(32)^, focal cortical thickening > 3 mm is the best malignancy indicator. In spite of being a late finding, absence of fatty hilum seems to be the most specific predictive factor for malignancy^(4,32)^. In the present sample, just one out of 23 cases of absent hilum was negative at histology (positive predictive value of 95.6%), suggesting the diagnosis of histoplasmosis.

Other predictive factors of malignancy have already been described by other authors, as follows: hypoechogenicity of the cortex and absence of central flow in the lymph node at Doppler^(4)^. Such factors were not evaluated in the present study. Lobulation or hypoechogenic asymmetry of the cortex, even < 3 mm, has already been reported as an early sign of malignancy^(13,33)^, although it was not identified in any of the 100 cases in the present study. Lymph node size is not a proven useful criterion to differentiate normal from abnormal lymph nodes^(4,7,9,24,33)^. However, the preliminary evaluation in the present study demonstrated that the transverse lymph node diameter ≥ 1 cm was the only factor indicating increased US-FNA sensitivity. Probably, such results reflect either the fact that in smaller compromised lymph nodes there was greater difficulty in performing US-FNA in the altered region, or that the changes were so precocious that they did not determine distortion in tumor morphology.

A limitation in the present study occurred due to the selection process of patients submitted to US-FNA, as only those presenting with some clinical or radiological suspicion of axillary compromising were included. The way the patients were selected may have been responsible for the high rates of the axillary tumor malignancy (68%), but nevertheless the authors do not believe that the inclusion of patients without any clinical or radiological suspicion can change the US-FNA sensitivity, since the present study suggests that only altered lymph nodes should be submitted to cytopathological evaluation.

## CONCLUSION

The decision about which patients should undergo axillary US still remains to be defined. Morphological lymph node alterations represent some os the main predictive factors of malignancy, and US is the preferred method for such evaluation, because of its low cost, wide accessibility and good reproducibility. With the results in mind, the authors propose that axillary US should be included in preoperative staging of all invasive breast cancer patients who are candidates to SLNB, regardless the tumor size and clinical evaluation of the axilla. Thus, it is possible to optimize the detection of axillary lymph nodes involvement. In order to maximize the US-FNA positiveness, it is advisable to perform it only in those patients presenting with morphologically altered lymph nodes.

**Figure 4:**
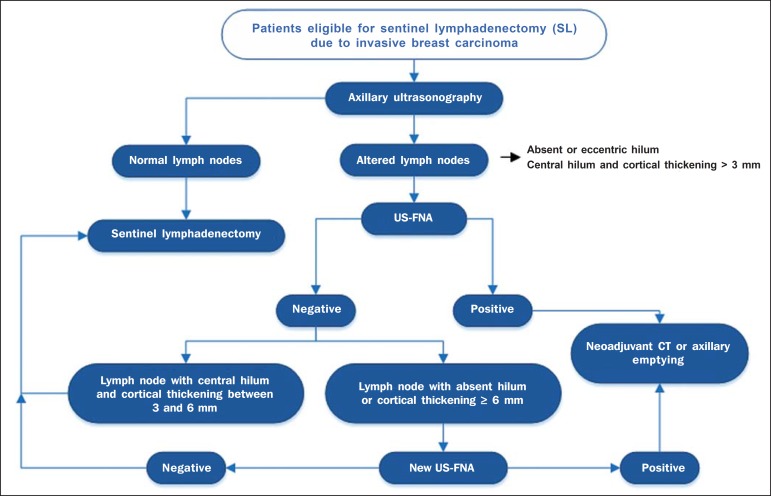
Algorithm for utilization of axillary ultrasonography and ultrasonography-guided fine needle aspiration (US-FNA) in pre-operative evaluation of invasive breast cancer.

The algorithm recommended by the authors is in Figure 4. The results confirm that the addition of FNA to US at a single moment can avoid more than 50% of the SLNBs, with a very low incidence of complications and, most probably, a significant reduction in costs and in the time interval until a definitive therapy is implemented.
